# Nutritional status and weight trajectories during radiotherapy in patients with cancer: a retrospective cohort study

**DOI:** 10.3389/fnut.2026.1957371

**Published:** 2026-09-16

**Authors:** Tong Liu, Shijin Wu

**Affiliations:** Department of Radiotherapy, The Fourth Clinical College of Henan Medical University, Xinxiang Central Hospital, Xinxiang, Henan, China

**Keywords:** cancer, malnutrition, nutritional status, nutritional support, prognostic nutritional index, radiotherapy, weight loss

## Abstract

**Background:**

Patients receiving radiotherapy are frequently malnourished, and weight loss can negatively impact treatment tolerance and clinical outcomes.

**Objective:**

This retrospective cohort study aimed to assess nutritional status, weight trajectories, and changes in the prognostic nutritional index (PNI) over time, during radiotherapy.

**Methodology:**

The medical records of 680 adult patients who initiated radiotherapy were reviewed retrospectively at a tertiary oncology center from 2023 to 2025. Patients were retained in the cohort regardless of subsequent treatment completion, provided they met predefined baseline eligibility criteria. Body weight, serum albumin and PNI were measured serially before, during and after radiotherapy. Weight and trajectory patterns of the PNI were described, toxicities associated with the treatment and nutritional interventions were evaluated, and multi-variable logistic regression models were constructed to identify factors associated with clinically marked weight loss (decrease of ≥5%).

**Results:**

Mean body weight decreased from 69.4 ± 14.8 kg at baseline to 65.8 ± 14.6 kg at treatment completion (*p* < 0.001). Clinically significant weight loss occurred in 44.6% of patients, while low PNI increased from 36.9 to 60.7%. Severe treatment-related toxicity, treatment interruption, hospitalization, and radiotherapy non-completion were significantly associated with weight loss and declining PNI trajectories (all *p* < 0.001). Nutritional support was associated with lower odds of weight loss (adjusted OR = 0.66, 95% CI: 0.46–0.94), although this finding should be interpreted cautiously because intervention timing was not consistently documented and causality cannot be established.

**Conclusion:**

During radiotherapy, nutritional deterioration is common and is closely linked to poor treatment outcomes. Early nutritional assessment and intervention can reduce weight loss and improve tolerance to therapy in high-risk patients.

## 1 Introduction

Cancer continues to be one of the top causes of death and disability around the world, and radiotherapy is an essential part of the treatment for a significant number of patients with solid cancers. Radiotherapy is beneficial for controlling the tumor locally and increasing the survival of the patient; however, it is often associated with toxicity, metabolic disturbances and decreased food consumption, thereby contributing to the risk of malnutrition and cachexia, sarcopenia and progressive weight loss (1). This has led to current clinical guidelines recommending routine nutritional assessment and nutritional management when needed during cancer treatment to optimize therapeutic outcomes (2).

Tumor burden, the inflammatory response, radiotherapy intensity, and the patient’s nutritional status before radiotherapy are complexly linked and affect nutritional status during radiotherapy. Clinical findings have shown strong links between nutritional biomarkers and feeding strategies, treatment tolerance, and outcomes for patients undergoing chemoradiotherapy (3). Similarly, in malnourished cancer patients, nutritional care has been linked to better clinical outcomes, confirming that it should be part of routine cancer care (4). Early consideration of nutritional interventions is also a crucial element of multidisciplinary oncology care, as it can help maintain nutritional status and improve treatment completion (5); nutrition interventions aimed at cancer cachexia may also reduce the metabolic decline associated with cancer treatment (6).

Nutrition interventions aimed at symptoms such as oral mucositis, dysphagia, xerostomia, anorexia and nausea are common side effects of radiotherapy. They can impact dietary intake and contribute to poor weight trajectories. Longitudinal studies have shown worsening nutritional status throughout treatment (7), and nutritional status has been linked to radiation-induced oral mucositis (8). Early nutritional intervention has been shown to minimize treatment-related complications and improve nutritional status during radiotherapy (9); individual nutrition management may help minimize adverse effects in patients undergoing pelvic irradiation (10). In addition, systematic evidence shows that nutrition intervention positively affects short-term clinical outcomes (11), and recent guidelines recommend standardized nutrition intervention throughout the peri-radiotherapy period (12). In addition, dietary counseling has been identified as a viable supportive intervention for radiation therapy (RT) (13).

Nutrient changes are not only prognostic for treatment tolerance. In radiation therapy settings, weight loss during treatment correlated with body composition changes and decreased long-term survival (14), and in real-world settings, objective measures of nutrition status decline during radiation therapy (15). Similarly, poor nutritional status has been associated with poor survival in cervical cancer patients undergoing radiotherapy (16), and recent studies have also demonstrated the value of a comprehensive nutritional assessment in predicting outcomes in this setting (17).

While previous investigations have shown the negative impact of radiotherapy (RT) on nutritional status and body weight in cancer patients, the majority have focused on certain types of cancer, investigated specific nutritional outcomes, or investigated the effectiveness of a nutritional intervention itself, but not on the impact of RT over time on the patients’ nutrition or body weight in a clinical setting. Thus, data on nutritional status and weight changes from a larger population of patients undergoing radiotherapy are scarce. Therefore, this retrospective cohort study compared nutritional status and weight changes during radiotherapy in cancer patients and investigated trends in nutritional parameters and body weight during radiotherapy.

## 2 Methodology

### 2.1 Study design and setting

This retrospective cohort study assessed nutritional status and growth patterns in adult cancer patients undergoing radiotherapy from 2023 to 2025. This study was conducted at a tertiary oncology center providing radiation oncology, medical oncology, clinical nutrition, and supportive care services. Clinical data were obtained from the electronic medical record, radiotherapy data, the laboratory information system, and nutrition assessment records.

The study aimed to describe weight loss and changes in nutritional parameters during radiotherapy, explore individual weight-trajectory patterns, and assess clinical and therapeutic parameters related to significant weight loss. The methodological framework was grounded in existing research that assessed weight changes associated with treatment, nutritional deterioration, and negative outcomes during radiotherapy, as well as trajectories of the prognostic nutritional index (PNI).

### 2.2 Study population and patient selection

The patients were selected from the institutional oncology and radiotherapy registry among those who initiated external-beam radiotherapy during the study period. Initially, 842 patient records were retrieved. After excluding 38 duplicate or incomplete records, 804 unique patient records were identified and screened for eligibility.

Patients were eligible if they were adults (aged ≥18 years) with a histologically confirmed cancer diagnosis who initiated external-beam radiotherapy, including definitive radiotherapy with or without concurrent chemotherapy, adjuvant radiotherapy, or neoadjuvant radiotherapy. Eligible patients had a baseline body weight documented within 14 days before radiotherapy initiation, at least two additional body weight measurements obtained during or after the treatment period when available, and adequate baseline clinical, treatment, and laboratory information for analysis.

Among the 804 screened records, 124 patients were excluded during eligibility assessment. These included 61 patients with missing baseline weight or insufficient serial weight measurements, 32 with incomplete clinical or treatment information, 18 with major non-cancer-related conditions likely to substantially affect body weight, and 13 who received radiotherapy outside the institution with incomplete treatment records. Importantly, discontinuation or non-completion of the planned radiotherapy course was not an exclusion criterion. Patients who initiated radiotherapy but subsequently discontinued treatment or received less than the planned radiation does remained eligible for cohort analysis, provided that the predefined baseline eligibility criteria were met. Finally, 680 patients were included in the final retrospective cohort. Figure 1 illustrates the patient selection process.

**Figure 1:**
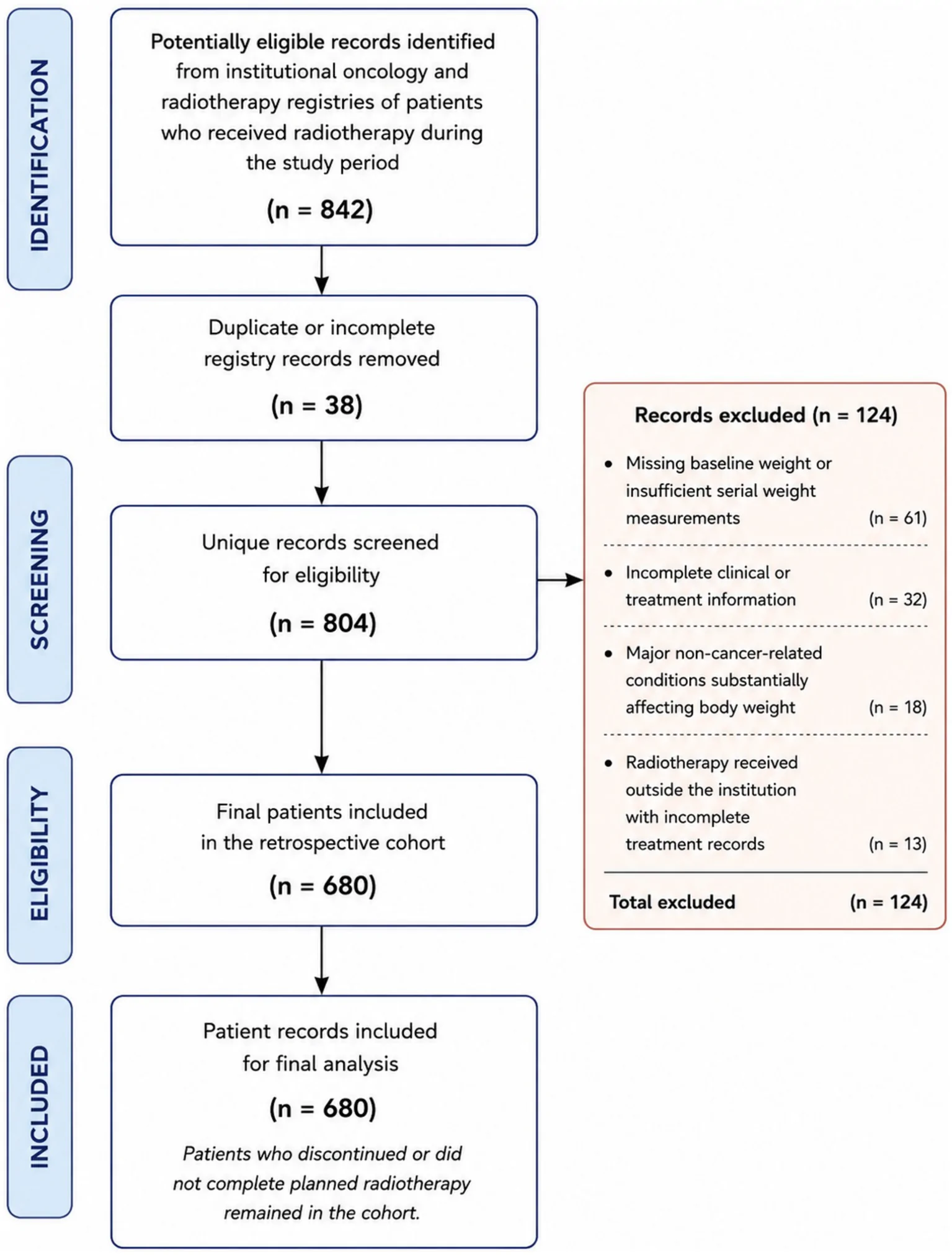
Patient selection flow diagram.

### 2.3 Data collection and study variables

Data were collected on a standardized data collection form. Demographic variables were age and sex. The clinical factors studied were primary tumor location, cancer stage, treatment planning, performance status and pertinent comorbidities. Tumor sites were classified into clinically relevant categories: head and neck, thoracic, upper gastrointestinal, pelvic, breast and others. The stage of the cancer was noted as per the oncology chart from the institution when radiotherapy was started, and was considered as a marker of disease severity. Performance status (PS) using the ECOG scale was not well recorded in the retrospective clinical records, and therefore was not a covariate in the primary analysis.

Radiotherapy-related factors included treatment intent, prescribed radiation dose, number of fractions, treatment duration, treatment interruption, and treatment completion. Other systemic anticancer therapy, such as concurrent chemotherapy, was also documented. Nutritional parameters included body weight, height, BMI, percentage weight change, serum albumin levels, absolute lymphocyte count, PNI, nutritional support, and dietitian referral.

At four predetermined assessment times before radiotherapy, during, at the end of radiotherapy, and early post-treatment follow-up, the body weight was measured. Weight was measured as part of routine care on calibrated weighing scales used in clinical practice in the oncology and radiotherapy departments. Wherever possible, serial measurements were taken for each patient with the same clinical weighing equipment or equivalent weighing equipment routinely calibrated. The body weight of the patients was recorded under standardized clinical conditions, which is normally without shoes and light clothing. If clothing removal was not possible due to clinical circumstances, clothing was not changed between serial assessments unless it could not be standardized. Measurements were typically taken at a planned outpatient or treatment appointment, mostly during the day, at the clinic. This was a retrospective study, and fasting information and the actual time of day were not always reported in routine clinical records, so they were not used as eligibility criteria or analytic adjustment variables.

The time between weight assessments was not standardized across all patients because treatment schedules varied by tumor location, therapeutic goals, prescribed dose, and fractionation regime. The actual radiotherapy duration was defined as the number of calendar days between the first and last radiotherapy fraction given. The interval between baseline, mid-treatment, and end-of-treatment weight assessments was determined by subtracting the dates of the previous weight assessments from the dates of the current assessment. The interval between the end-of-treatment and early follow-up weight assessments was determined by subtracting the dates of the early follow-up weight assessments from the end of treatment weight assessments. Measurements were aligned for longitudinal analyses according to the specified treatment phases, not equal calendar intervals, because treatment periods varied.

Baseline weight was taken within 14 days prior to the first radiotherapy session. Mid-treatment weight was defined as the weight taken closest to the middle of the radiotherapy course. Patients were considered to have completed treatment at the end of the last radiotherapy session or within 7 days. Weight measured 4–8 weeks after radiotherapy was considered early post-treatment weight. If more than one weight measurement occurred within an assessment window, we used the one closest to the predetermined time point for analysis.

### 2.4 Nutritional status and weight assessment

The BMI was computed as the body weight (kg) divided by height squared (m^2^) (18). Underweight status was defined as BMI < 18.5 kg/m^2^, normal weight as BMI ranging from 18.5 to 24.9 kg/m^2^, overweight as BMI ranging from 25.0 to 29.9 kg/m^2^ and obese as BMI ≥ 30.0 kg/m^2^.

The weight change was expressed as a percentage using the equation below:

$$\begin{array}{l} \text{Percentage weight change}=\\ {}[(\text{follow}-\mathrm{up}\;\text{weight}–\text{baseline weight})/\text{baseline weight}]\times 100. \end{array}$$

Weight loss of ≥5% from baseline body weight was considered clinically significant weight loss during radiotherapy. Critical weight loss was defined as a loss of ≥5% during radiotherapy or >10% over a longer observation period. The change in body weight within ±2% of the original weight was defined as weight stability (1, 2).

Nutritional status was determined using available anthropometric, biochemical, and clinical nutrition data. Hypoalbuminemia was considered to be serum albumin <35 g/L. Nutritional risk and nutritional intervention needs were identified, when available, based on documented clinical nutrition assessment, such as Patient-Generated Subjective Global Assessment (PG-SGA) (19, 20). A PG-SGA score of ≥9 indicated a critical need for nutritional intervention (19).

### 2.5 Weight and PNI trajectory classification

The longitudinal weight trajectories were assessed by repeated weight measurements before, during and after radiotherapy. Clinically relevant trajectory groups included stable weight, gradual weight loss, marked weight loss, and weight recovery. A stable trajectory was defined as a weight change of ±2% of baseline. A progressive loss trajectory was defined as progressive weight loss of less than 5%. A marked loss trajectory was determined as ≥5% loss from baseline at radiotherapy. A recovery course was characterized by initial weight loss followed by partial weight regain in the early post-treatment follow-up phase.

The following formula was used to calculate PNI (21):

$$\begin{array}{l} \mathrm{PNI}=\text{serum albumin}\;(g/L)\\ +0.005\times \text{total lymphocyte count}\;(/{\mathrm{mm}}^{3}). \end{array}$$

Laboratory data were available to assess PNI at baseline and at later time points. A PNI < 45 was deemed an indicator of greater nutritional vulnerability. PNI was also measured as a continuous variable, as clinically meaningful cut-off points can vary by tumor type and treatment features. Patterns of PNI were determined by comparing the changes over time as stable/improving, mildly declining or markedly declining.

### 2.6 Clinical outcomes and treatment-related toxicity

The incidence of treatment-related toxicities was collected from clinical records and graded using the Common Terminology Criteria for Adverse Events (CTCAE), version 5.0. (22). Oral mucositis, dysphagia, odynophagia, nausea, vomiting, diarrhea, anorexia, and fatigue were the outcomes of interest. The severity of the toxicity related to treatment was defined as >grade 3 in CTCAE.

An unplanned interruption of at least three consecutive treatment days was considered a treatment interruption, and an interruption of more than five treatment days was considered a prolonged treatment interruption. Radiotherapy completion was defined as delivery of ≥95% of the prescribed radiation dose. Toxicity, resulting in hospitalization due to treatment related complications, nutritional compromise, or dehydration was also noted during the course of radiation therapy.

Clinically significant weight loss was the primary outcome during radiotherapy. Secondary outcomes were poor weight and PNI trajectories, treatment-related severe toxicity, treatment interruption, hospitalization and non-completion of the intended course of radiotherapy.

### 2.7 Operational definitions

A body weight recorded within 14 days before initiation of radiotherapy was considered baseline weight. Clinically significant weight loss was defined as ≥5% from baseline weight measured during radiotherapy, and critical weight loss as ≥5% during radiotherapy or ≥10% during the longer follow-up (23). For secondary analysis of weight loss severity, patients were divided into three groups: <5% BW loss, 5–10% BW loss, and >10% BW loss, from baseline weight during radiotherapy. The <5% category was defined as having experienced no clinically significant weight loss, the 5–10% represented a moderate level of weight loss and the >10% was a more severe level of weight loss. Weight stability was determined by a change of ±2% from baseline.

Low PNI was defined as <45 (24). Underweight was defined as BMI < 18.5 kg/m2 and hypoalbuminemia as <35 g/L (18, 25). Treatment-related toxicity was graded according to the CTCAE criteria and considered severe if it reached grade ≥3 (26). Radiotherapy completion was defined as receiving ≥95% of the prescribed dose, and treatment non-completion was defined as receiving <95% of the prescribed dose. Cancer stage was categorized according to the stage documented at the time of radiotherapy initiation and was included as a categorical measure of disease severity.

Earlier nutritional support was defined as a nutrition assessment by the dietitian and/or early nutritional intervention (oral or enteral nutrition) before radiotherapy initiation or early in the radiotherapy period (when it could be identified). A comprehensive timing analysis of early versus delayed nutritional consultation or intervention was not possible because exact timing was not systematically recorded for all patients.

Radiotherapy completion and non-completion: Radiotherapy completion was defined as receiving ≥95% of the prescribed radiation dose. Non-completion of radiotherapy was considered when less than 95% of the planned radiotherapy dose was delivered due to toxicity from radiotherapy, clinical deterioration, patient choice, disease progression or due to other documented clinical reasons. Patients who did not complete radiotherapy were included in the analytical cohort and analysed based on the treatment course they received.

### 2.8 Statistical analysis

IBM SPSS Statistics for Windows version 29.0 (IBM Corp., Armonk, NY, USA) was used for statistical analysis. The Shapiro–Wilk normality test was used to determine the normality of continuous variables, and distribution plots were observed. Normally distributed data were expressed as mean ± standard deviation (SD), and non-normally distributed data were expressed as median and interquartile range (IQR). Categorical variables were reported as frequencies and percentages. Demographic, clinical, treatment-related and nutritional baseline characteristics were compared between the weight-trajectory groups. Independent samples *t*-test or one-way analysis of variance (ANOVA) was used for variables with normal distribution, and the Mann–Whitney *U* or Kruskal–Wallis test was used for variables that do not have a normal distribution. The chi-square test and/or Fisher’s exact test were used to compare categorical variables, as appropriate.

The secondary weight-loss severity analysis compared treatment-related outcomes between weight loss categories (<5%, 5–10 and >10%) using the chi-square test or Fisher’s exact test, as appropriate. The incidence of treatment-related adverse outcomes was evaluated for linear trend, as appropriate, within weight-loss categories. Changes in body weight, percentage weight change, serum albumin, and PNI were measured with repeated-measures analysis. If measurements were repeated but the number of measurements per patient differed, a linear mixed-effects model was used. Within-patient correlation was accounted for by including patient identification as a random effect, and time as a fixed effect.

Multivariable and univariable logistic regression analyses were conducted to assess the relationship between baseline characteristics and treatment planning factors with clinically meaningful weight loss (WL ≥ 5%) during radiotherapy. The primary multivariable analysis used only variables collected before or at radiotherapy initiation, including age, sex, baseline BMI, pre-treatment weight loss, tumor location, cancer stage, treatment intent, planned radiotherapy dose, planned concurrent chemotherapy, baseline albumin, and baseline PNI. Cancer stage was included because disease severity may influence nutritional status and treatment tolerance. ECOG performance status was not included because it was not consistently documented in the retrospective records. Longitudinal variables during radiotherapy such as severe toxicity related to the radiotherapy treatment and PNI trajectory were not selected as baseline predictors because they may be associated with, may mediate, or be a consequence of weight loss.

Before analysis, the extent and pattern of missingness was assessed. In the case of longitudinal nutritional variables all observations, regardless of the number of times the variable was measured, were kept at each assessment time point. No patients were excluded from the analytical cohort just because they did not have any measurements after radiotherapy. The number of observations may therefore vary between assessment time points, especially at the end of treatment and at early follow-up. If the number of measurements per patient was unequal and it was assumed that the measurements occurred at random, a linear mixed-effects model was used. Because exact dates of nutritional consultation or intervention were not consistently available, formal comparison of weight trajectories according to early versus delayed nutritional intervention was not performed. Nutritional-support findings were therefore analyzed descriptively and interpreted as observational associations. For other variables, complete case analysis was performed when missingness was low. For variables with a substantial amount of missing information, multiple imputation was considered when the missing-at-random assumption was judged reasonable. Two-sided statistical tests were performed, and *p* < 0.05 was regarded as statistically significant.

### 2.9 Ethical considerations

This research was approved by the Ethics Committee of Xinxiang Central Hospital: Approval Number: 20260610. The study was conducted in accordance with the principles of the Declaration of Helsinki and its amendments (27). Patient confidentiality was preserved during the collection, management, analysis and reporting of data. All extracted data were anonymised and shared only with authorized members of the research team.

## 3 Results

### 3.1 Patient baseline characteristics

The final analytical cohort comprised 680 patients. The mean age was 57.8 ± 12.9 years, and 414 patients (60.9%) were male. Most patients were aged 50–69 years (*n* = 371, 54.6%), followed by those aged ≥70 years (*n* = 176, 25.9%). Head and neck malignancies represented the largest tumor group (*n* = 196, 28.8%), followed by thoracic cancers (*n* = 137, 20.1%) and upper gastrointestinal cancers (*n* = 112, 16.5%). Stage III disease was the most frequently observed disease stage (*n* = 281, 41.3%), while 255 patients (37.5%) had stage II disease.

At baseline, the mean body weight was 69.4 ± 14.8 kg, and the mean BMI was 24.9 ± 4.6 kg/m^2^. A total of 84 patients (12.4%) were underweight, whereas 228 (33.5%) were overweight and 119 (17.5%) had obesity. Clinically relevant pre-treatment weight loss of ≥5% was documented in 143 patients (21.0%). The mean baseline serum albumin concentration was 38.1 ± 5.0 g/L, and hypoalbuminemia was present in 132 patients (19.4%). The mean baseline PNI was 46.7 ± 6.1, with 251 patients (36.9%) having a PNI < 45. Concurrent chemotherapy was administered to 402 patients (59.1%). Definitive radiotherapy was the most common treatment approach (*n* = 371, 54.6%), followed by adjuvant radiotherapy (*n* = 214, 31.5%) and neoadjuvant radiotherapy (*n* = 95, 14.0%). The median prescribed radiation dose was 62 Gy (IQR: 54–66 Gy). Table 1 presents the baseline demographic, clinical, treatment, and nutritional characteristics of the study cohort.

**Table 1:** Baseline demographic, clinical, treatment, and nutritional characteristics of the study cohort (*n* = 680).

Characteristic	Value
Age, years, mean ± SD	57.8 ± 12.9
<50 years	133 (19.6)
50–69 years	371 (54.6)
≥70 years	176 (25.9)
Sex	
Male	414 (60.9)
Female	266 (39.1)
Primary tumor site	
Head and neck	196 (28.8)
Thoracic	137 (20.1)
Upper gastrointestinal	112 (16.5)
Pelvic	104 (15.3)
Breast	71 (10.4)
Other	60 (8.8)
Cancer stage	
Stage I	71 (10.4)
Stage II	255 (37.5)
Stage III	281 (41.3)
Stage IV, limited/oligometastatic	73 (10.7)
Treatment intent	
Definitive	371 (54.6)
Adjuvant	214 (31.5)
Neoadjuvant	95 (14.0)
Concurrent chemotherapy	402 (59.1)
Prescribed radiation dose, Gy, median (IQR)	62 (54–66)
Actual radiotherapy duration, days	42 (36–49)
Observed range, days	18–71
Baseline nutritional characteristics	
Body weight, kg, mean ± SD	69.4 ± 14.8
BMI, kg/m^2^, mean ± SD	24.9 ± 4.6
Underweight, BMI < 18.5 kg/m^2^	84 (12.4)
Normal BMI, 18.5–24.9 kg/m^2^	249 (36.6)
Overweight, BMI 25.0–29.9 kg/m^2^	228 (33.5)
Obesity, BMI ≥ 30.0 kg/m^2^	119 (17.5)
Pre-treatment weight loss ≥5%	143 (21.0)
Serum albumin, g/L, mean ± SD	38.1 ± 5.0
Hypoalbuminemia, <35 g/L	132 (19.4)
Baseline PNI, mean ± SD	46.7 ± 6.1
Low baseline PNI, <45	251 (36.9)
Dietitian referral before radiotherapy	238 (35.0)
Nutritional supplementation at baseline	171 (25.1)

Values are presented as *n* (%) unless otherwise stated. Continuous non-normally distributed variables are presented as median (IQR). BMI, body mass index; PNI, prognostic nutritional index; SD, standard deviation; IQR, interquartile range.

### 3.2 Longitudinal changes in body weight and nutritional indicators

The median time for delivered radiotherapy was 42 days (IQR: 36–49 days; range: 18–71 days). The median time from baseline to the mid-treatment assessment was 21 days (IQR: 18–25 days) and the median time from baseline to the end-of-treatment assessment was 42 days (IQR: 36–49 days). Median early post-treatment follow up time was 42 days (IQR: 35–54 days) after the completion of radiotherapy. The timing of assessment on the calendar was different between tumor sites and treatment regimens, but longitudinal measurements were analyzed according to the predefined clinical treatment phases. Body weight decreased progressively throughout radiotherapy. The mean weight declined from 69.4 ± 14.8 kg at baseline to 67.9 ± 14.7 kg at mid-treatment, 65.8 ± 14.6 kg at treatment completion, and 65.2 ± 14.5 kg during early post-treatment follow-up. The mean percentage weight change was −2.2% at mid-treatment, −5.2% at treatment completion, and −6.0% at early follow-up. The overall reduction in body weight across the assessment period was statistically significant (*p* < 0.001).

The proportion of patients with clinically significant weight loss increased from 8.7% at mid-treatment to 44.6% at treatment completion and 51.8% at early follow-up. Critical weight loss was observed in 27.5% of patients by the end of radiotherapy and increased to 34.1% during early post-treatment follow-up. Nutritional biomarkers showed a similar pattern. Mean serum albumin declined from 38.1 ± 5.0 g/L at baseline to 34.8 ± 5.3 g/L at treatment completion. The prevalence of hypoalbuminemia increased from 19.4 to 46.0%. Mean PNI decreased from 46.7 ± 6.1 at baseline to 42.8 ± 6.4 at treatment completion, while the proportion of patients with PNI < 45 increased from 36.9 to 60.7%. Longitudinal changes in body weight and nutritional indicators during and after radiotherapy are summarized in Table 2.

**Table 2:** Longitudinal changes in body weight and nutritional indicators during and after radiotherapy (*n* = 680).

Variable	Baseline	Mid-radiotherapy	End of radiotherapy	Early follow-up	*p*-value
Body weight, kg	69.4 ± 14.8	67.9 ± 14.7	65.8 ± 14.6	65.2 ± 14.5	<0.001
Change from baseline, kg	–	−1.5 ± 1.8	−3.6 ± 3.2	−4.2 ± 4.0	<0.001
Percentage weight change	–	−2.2 ± 2.6	−5.2 ± 4.5	−6.0 ± 5.6	<0.001
Clinically significant weight loss, ≥5%	0	59 (8.7)	303 (44.6)	352 (51.8)	<0.001
Critical weight loss	0	21 (3.1)	187 (27.5)	232 (34.1)	<0.001
Serum albumin, g/L	38.1 ± 5.0	36.7 ± 5.2	34.8 ± 5.3	35.2 ± 5.4	<0.001
Hypoalbuminemia, <35 g/L	132 (19.4)	228 (33.5)	313 (46.0)	291 (42.8)	<0.001
PNI	46.7 ± 6.1	44.8 ± 6.3	42.8 ± 6.4	43.2 ± 6.6	<0.001
Low PNI, <45	251 (36.9)	348 (51.2)	413 (60.7)	401 (59.0)	<0.001

Continuous variables are presented as mean ± SD, and categorical variables are presented as *n* (%). *p*-values were derived from repeated-measures analyses. PNI, prognostic nutritional index.

Mean body weight decreased progressively from baseline through the completion of radiotherapy and remained below baseline during early post-treatment follow-up, as illustrated in Figure 2.

**Figure 2:**
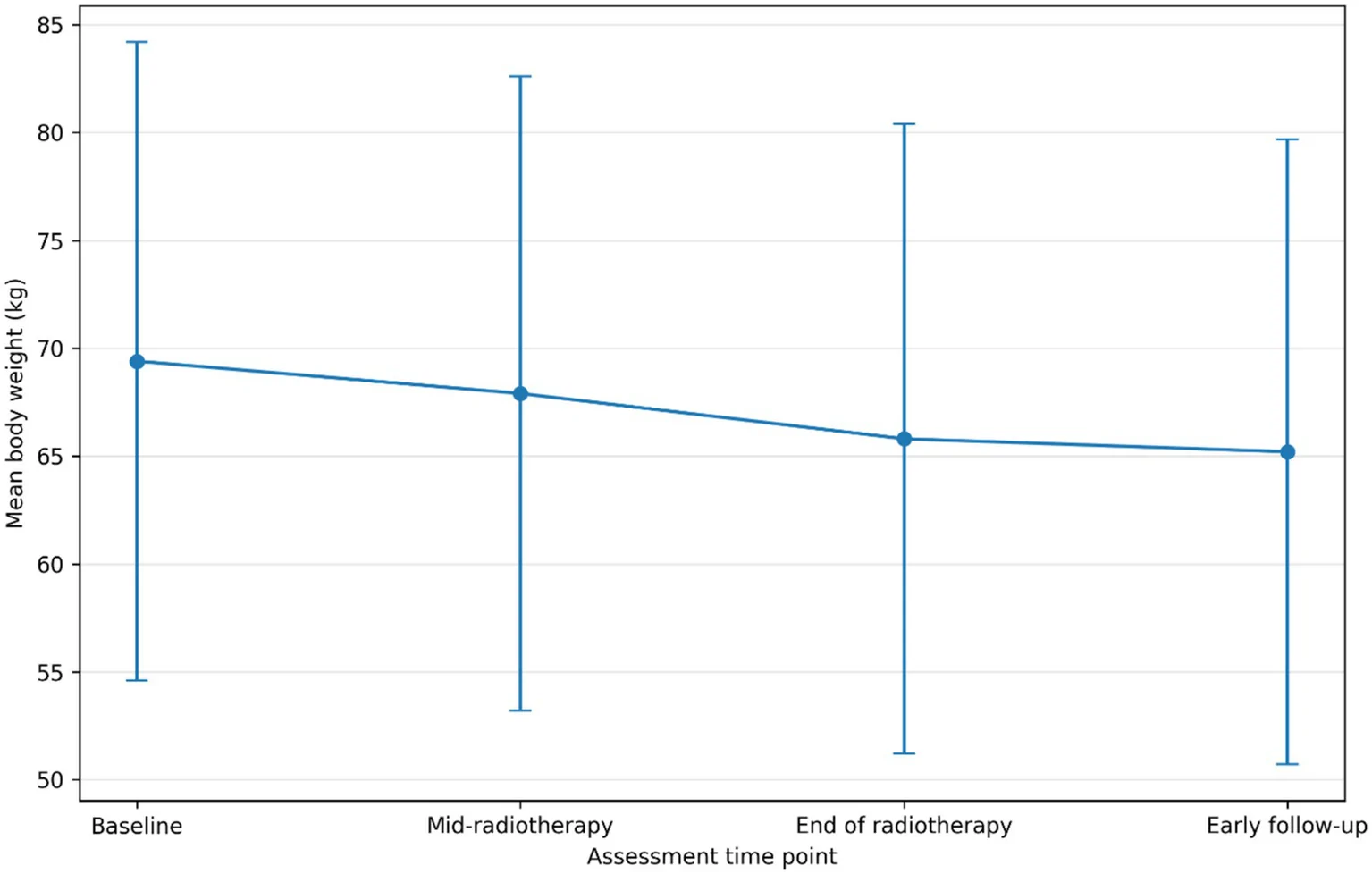
Longitudinal changes in mean body weight during and after radiotherapy.

Mean body weight declined from baseline to mid-radiotherapy and continued to decrease at treatment completion and early post-treatment follow-up. Data are expressed as mean body weight (kg). Standard deviations are shown as error bars. Baseline weight was determined within 14 days before the start of radiotherapy, and early follow-up weight was taken about 4–8 weeks after radiotherapy ended. Assessment points represent predefined clinical treatment phases; calendar intervals between assessments varied across patients because radiotherapy schedules and treatment duration differed.

### 3.3 Weight-trajectory categories and clinical characteristics

Four longitudinal weight-trajectory patterns were identified. A stable-weight trajectory was observed in 172 patients (25.3%), gradual weight loss in 185 patients (27.2%), marked weight loss in 245 patients (36.0%), and weight recovery after an initial decline in 78 patients (11.5%). Patients with marked weight loss had a lower baseline BMI and lower baseline albumin and PNI values than those with stable weight. They were also more likely to have head and neck or upper gastrointestinal cancers, receive concurrent chemotherapy, experience severe treatment-related toxicity, and require enteral nutritional support. The marked-loss group had the highest frequency of treatment interruption and hospitalization. The baseline and treatment characteristics according to weight-trajectory category are presented in Table 3.

**Table 3:** Baseline and treatment characteristics according to weight-trajectory category.

Characteristic	Stable (*n* = 172)	Gradual loss (*n* = 185)	Marked loss (*n* = 245)	Recovery (*n* = 78)	*p*-value
Age, years	58.6 ± 12.7	57.4 ± 13.1	56.9 ± 12.8	59.0 ± 12.5	0.42
Male sex	100 (58.1)	111 (60.0)	160 (65.3)	43 (55.1)	0.18
Baseline BMI, kg/m^2^	26.0 ± 4.5	25.1 ± 4.4	23.6 ± 4.7	25.4 ± 4.3	<0.001
Baseline weight loss ≥5%	20 (11.6)	34 (18.4)	78 (31.8)	11 (14.1)	<0.001
Albumin, g/L	39.5 ± 4.7	38.4 ± 4.8	36.5 ± 5.3	38.6 ± 4.9	<0.001
Baseline PNI	49.0 ± 5.7	47.3 ± 5.8	43.8 ± 6.4	47.1 ± 5.9	<0.001
Head and neck cancer	35 (20.3)	52 (28.1)	89 (36.3)	20 (25.6)	0.002
Upper gastrointestinal cancer	20 (11.6)	28 (15.1)	53 (21.6)	11 (14.1)	0.01
Concurrent chemotherapy	80 (46.5)	106 (57.3)	175 (71.4)	41 (52.6)	<0.001
Severe toxicity, grade ≥3	19 (11.0)	39 (21.1)	103 (42.0)	17 (21.8)	<0.001
Enteral nutritional support	8 (4.7)	18 (9.7)	69 (28.2)	11 (14.1)	<0.001
Treatment interruption	12 (7.0)	25 (13.5)	67 (27.3)	11 (14.1)	<0.001
Hospitalization during treatment	9 (5.2)	17 (9.2)	55 (22.4)	8 (10.3)	<0.001

Values are presented as mean ± SD or *n* (%). The marked-loss trajectory was defined as ≥5% weight reduction from baseline during radiotherapy.

### 3.4 Prognostic nutritional index trajectories

Three PNI trajectory patterns were identified: stable or improving PNI in 219 patients (32.2%), mildly declining PNI in 251 patients (36.9%), and markedly declining PNI in 210 patients (30.9%). Patients with a markedly declining PNI trajectory was associated with greater concurrent weight loss and also had higher frequencies of treatment interruption, hospitalization, and radiotherapy non-completion than patients with stable or improving PNI trajectories (Table 4).

**Table 4:** Clinical outcomes according to PNI trajectory category.

Outcome	Stable/improving (*n* = 219)	Mild decline (*n* = 251)	Marked decline (*n* = 210)	*p*-value
Baseline PNI	48.9 ± 5.6	46.8 ± 5.7	44.1 ± 6.3	<0.001
End-treatment PNI	48.2 ± 5.9	42.9 ± 5.8	36.8 ± 6.1	<0.001
Mean weight change, %	−2.7 ± 3.5	−5.1 ± 4.2	−8.2 ± 5.1	<0.001
Clinically significant weight loss	45 (20.5)	117 (46.6)	141 (67.1)	<0.001
Hypoalbuminemia at treatment completion	54 (24.7)	121 (48.2)	138 (65.7)	<0.001
Severe treatment-related toxicity	28 (12.8)	65 (25.9)	85 (40.5)	<0.001
Severe oral mucositis^*^	14 (14.6)	32 (27.8)	47 (48.5)	<0.001
Treatment interruption	17 (7.8)	42 (16.7)	56 (26.7)	<0.001
Hospitalization	13 (5.9)	29 (11.6)	47 (22.4)	<0.001
Radiotherapy non-completion	5 (2.3)	12 (4.8)	22 (10.5)	0.002

^*^Severe oral mucositis was assessed among patients with head and neck malignancies.

PNI, prognostic nutritional index. Values are presented as mean ± SD or *n* (%). A markedly declining PNI trajectory represented a sustained reduction across serial assessments. PNI trajectory was derived from serial measurements obtained during radiotherapy, it was interpreted as a longitudinal marker of concurrent nutritional deterioration and was not included as a predictor in the primary multivariable model of clinically significant weight loss. Radiotherapy non-completion was defined as delivery of <95% of the prescribed radiation dose. Patients with radiotherapy non-completion remained in the analytical cohort.

### 3.5 Nutritional support and weight outcomes

A total of 238 patients (35.0%) received dietitian consultation before radiotherapy, while 171 patients (25.1%) initiated oral nutritional supplementation at baseline. During treatment, nutritional support was intensified in 298 patients (43.8%). Oral nutritional supplementation was provided to 223 patients (32.8%), and enteral nutritional support was required in 106 patients (15.6%). Feeding-tube support was more common among patients with head and neck and upper gastrointestinal cancers. Patients with documented nutritional support during the early treatment period had a lower observed frequency of marked weight loss. Because the exact timing of nutritional intervention was not consistently recorded, a formal analysis comparing early versus delayed intervention and subsequent weight trajectory was not performed. Nutritional support practices and associated clinical outcomes are presented in Table 5.

**Table 5:** Nutritional support and clinical outcomes during radiotherapy.

Variable	Early nutritional support (*n* = 238)	Delayed/no early support (*n* = 442)	*p*-value
Baseline BMI, kg/m^2^	24.2 ± 4.7	25.3 ± 4.5	0.004
Baseline PNI	45.5 ± 6.2	47.3 ± 5.9	<0.001
Mean weight change at treatment completion, %	−4.6 ± 4.2	−5.5 ± 4.6	0.01
Marked weight-loss trajectory	68 (28.6)	177 (40.0)	0.003
Severe treatment-related toxicity	54 (22.7)	124 (28.1)	0.12
Treatment interruption	31 (13.0)	84 (19.0)	0.04
Hospitalization	23 (9.7)	66 (14.9)	0.05
Radiotherapy completion ≥95%	228 (95.8)	407 (92.1)	0.07

Early nutritional support as dietitian assessment and/or initiation of oral or enteral nutritional intervention before radiotherapy initiation or within the first predefined treatment interval, before the occurrence of the primary ≥5% weight-loss outcome whenever documented. Values are presented as mean ± SD or *n* (%).

### 3.6 Treatment-related toxicity and clinical outcomes

Treatment-related toxicity was common during radiotherapy. At least one moderate-to-severe treatment-related adverse event was documented in 431 patients (63.4%), while severe toxicity (CTCAE grade ≥3) occurred in 178 patients (26.2%). The most frequent severe toxicities were mucositis, dysphagia, anorexia, and gastrointestinal symptoms. Treatment interruption and radiotherapy non-completion were evaluated among all eligible patients who initiated radiotherapy and remained within the final analytical cohort. Patients with clinically significant weight loss had higher rates of treatment interruption, prolonged interruption, hospitalization, and radiotherapy non-completion than those with weight loss of <5% (Table 6).

**Table 6:** Treatment-related outcomes according to clinically significant weight loss.

Outcome	Weight loss <5% (*n* = 377)	Weight loss ≥5% (*n* = 303)	*p*-value
Any moderate-to-severe toxicity	207 (54.9)	224 (73.9)	<0.001
Severe toxicity, grade ≥3	67 (17.8)	111 (36.6)	<0.001
Severe oral mucositis^*^	34 (20.9)	59 (42.1)	<0.001
Severe dysphagia^†^	27 (16.6)	58 (41.4)	<0.001
Severe anorexia	43 (11.4)	72 (23.8)	<0.001
Treatment interruption	39 (10.3)	76 (25.1)	<0.001
Prolonged interruption >5 days	16 (4.2)	41 (13.5)	<0.001
Hospitalization	28 (7.4)	61 (20.1)	<0.001
Radiotherapy non-completion	10 (2.7)	29 (9.6)	<0.001

^*^Assessed among patients with head and neck malignancies.

^†^Assessed among patients with head and neck, thoracic, and upper gastrointestinal malignancies.

Radiotherapy non-completion was defined as delivery of <95% of the prescribed radiation dose. Patients with radiotherapy non-completion remained in the analytical cohort. Clinically significant weight loss was defined as a reduction of ≥5% from baseline body weight during radiotherapy. Values are presented as *n* (%).

### 3.7 Baseline factors associated with clinically significant weight loss

Multivariable logistic regression identified several independent factors associated with clinically significant weight loss during radiotherapy. Pre-treatment weight loss, low baseline BMI, low baseline PNI, advanced cancer stage, head & neck cancer, upper gastrointestinal cancer and concurrent chemotherapy were all independently associated with higher odds of clinically significant weight loss. Patients with Stage III disease were more likely to have clinically significant weight loss (adjusted OR 1.63, 95% CI 1.15–2.32; *p* = 0.006) and Stage IV disease was associated with greater odds (adjusted OR 1.91, 95% CI 1.23–2.97; *p* = 0.004) when compared with patients with Stage I-II disease. Age, sex were not significant predictors. Results of multivariable logistic regression analysis are shown in Table 7.

**Table 7:** Baseline and treatment-planning factors independently associated with clinically significant weight loss during radiotherapy.

Predictor	Adjusted OR	95% CI	*p*-value
Age ≥70 years	1.16	0.80–1.68	0.44
Male sex	1.12	0.79–1.58	0.53
Baseline BMI < 18.5 kg/m^2^	1.68	1.04–2.72	0.03
Pre-treatment weight loss ≥5%	2.41	1.63–3.57	<0.001
Cancer stage: Stage I–II	Reference	–	–
Stage III vs. Stage I–II	1.63	1.15–2.32	0.006
Stage IV vs. Stage I–II	1.91	1.23–2.97	0.004
Baseline PNI < 45	1.74	1.22–2.48	0.002
Head and neck cancer	2.16	1.42–3.28	<0.001
Upper gastrointestinal cancer	1.79	1.12–2.87	0.02
Planned concurrent chemotherapy	1.92	1.34–2.75	<0.001

Adjusted odds ratios were estimated using multivariable logistic regression. The outcome was clinically significant weight loss, defined as a ≥ 5% reduction in body weight from baseline during radiotherapy. Only variables available before or at radiotherapy initiation were included in the primary multivariable model to preserve temporal ordering. Cancer stage was entered as a categorical variable, with Stage I–II as the reference category. OR, odds ratio; CI, confidence interval; BMI, body mass index; PNI, prognostic nutritional index.

Figure 3 presents the adjusted associations between demographic, nutritional, tumor-related, and treatment-related factors and clinically significant weight loss.

**Figure 3:**
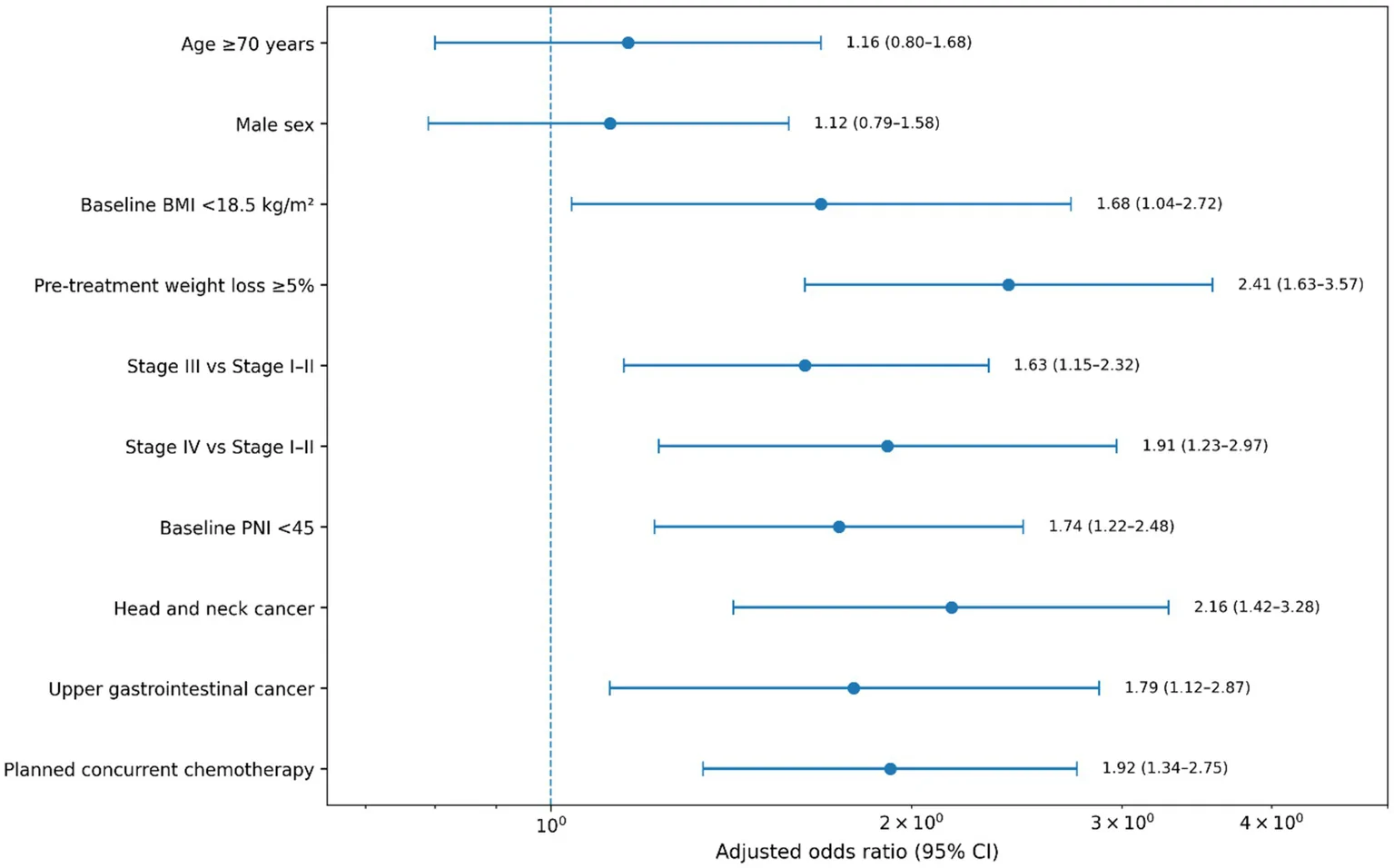
Baseline and treatment-planning factors associated with clinically significant weight loss during radiotherapy.

The forest plot presents adjusted odds ratios and 95% confidence intervals from the primary multivariable logistic regression model. Only variables available before or at radiotherapy initiation were included to preserve temporal ordering.

### 3.8 Subgroup analysis according to tumor site

Nutritional outcomes differed across primary tumor sites. The highest mean weight loss (−7.1 ± 5.4%), prevalence of clinically significant weight loss (60.7%), PNI decline (39.8%), severe toxicity (36.2%) and enteral nutritional support (29.1%) occurred in patients with head and neck cancer. Similar poor nutritional outcomes were observed in patients with upper gastrointestinal cancer. In contrast, breast cancer patients had the lowest rates of weight loss (18.3%), severe PNI decline (16.9%), severe toxicity (11.3%) and enteral nutritional support (1.4%). Nutritional outcomes by primary tumor site are summarized in Table 8.

**Table 8:** Nutritional outcomes according to primary tumor site.

Tumor site	Mean weight change, %	Weight loss ≥5%	Marked PNI decline	Severe toxicity	Enteral support
Head and neck (*n* = 196)	−7.1 ± 5.4	119 (60.7)	78 (39.8)	71 (36.2)	57 (29.1)
Thoracic (*n* = 137)	−5.3 ± 4.7	62 (45.3)	40 (29.2)	34 (24.8)	16 (11.7)
Upper gastrointestinal (*n* = 112)	−6.6 ± 5.1	64 (57.1)	39 (34.8)	37 (33.0)	27 (24.1)
Pelvic (*n* = 104)	−3.8 ± 3.9	33 (31.7)	26 (25.0)	18 (17.3)	4 (3.8)
Breast (*n* = 71)	−2.4 ± 3.1	13 (18.3)	12 (16.9)	8 (11.3)	1 (1.4)
Other (*n* = 60)	−3.5 ± 4.0	12 (20.0)	15 (25.0)	10 (16.7)	1 (1.7)
Overall (*n* = 680)	−5.2 ± 4.5	303 (44.6)	210 (30.9)	178 (26.2)	106 (15.6)

Values are presented as mean ± SD or *n* (%). Weight change represents the percentage change from baseline to completion of radiotherapy. PNI, prognostic nutritional index.

### 3.9 Treatment-related outcomes according to weight-loss severity

In terms of severity of weight loss, increasing treatment-related morbidity was seen with higher weight loss. There were no differences in moderate or severe toxicity between the three groups of patients with <5% weight loss, 5–10% weight loss and >10% weight loss, respectively (54.9% vs. 65.0% vs. 98.8%, *p* < 0.001). This was also true for grade ≥3 toxicity, which rose from 17.8% in the patients with <5% weight loss to 31.4 and 51.3% in the patients with 5–10 and >10% weight loss, respectively (*p* < 0.001). Treatment interruption, prolonged interruption, hospitalization and radiotherapy non-completion also increased significantly with the severity of the weight loss (all *p* < 0.001). Treatment outcomes by weight loss severity are shown in Table 9.

**Table 9:** Treatment-related outcomes according to weight-loss severity during radiotherapy.

Treatment-related outcome	<5% (*n* = 377)	5–10% (*n* = 223)	>10% (*n* = 80)	*p*-value
Any moderate-to-severe toxicity	207 (54.9)	145 (65.0)	79 (98.8)	<0.001
Grade ≥3 toxicity	67 (17.8)	70 (31.4)	41 (51.3)	<0.001
Severe oral mucositis^*^	34 (20.9)	36 (35.0)	23 (48.9)	<0.001
Severe dysphagia^†^	27 (16.6)	35 (34.3)	23 (48.9)	<0.001
Severe anorexia	43 (11.4)	45 (20.2)	27 (33.8)	<0.001
Treatment interruption	39 (10.3)	48 (21.5)	28 (35.0)	<0.001
Prolonged interruption >5 days	16 (4.2)	25 (11.2)	16 (20.0)	<0.001
Hospitalization	28 (7.4)	38 (17.0)	23 (28.8)	<0.001
Radiotherapy non-completion	10 (2.7)	18 (8.1)	11 (13.8)	<0.001

^*^Assessed among patients with head and neck malignancies.

^†^Assessed among clinically relevant tumor-site groups.

Values are presented as *n* (%). Weight-loss categories were defined according to the maximum percentage reduction from baseline body weight during radiotherapy. *p*-values represent comparisons across the three weight-loss categories.

## 4 Discussion

In the present study, even though the mean BMI appeared to be normal, many patients began radiotherapy with locally advanced disease and with pre-existing nutritional compromise, such as weight loss, hypoalbuminemia and low PNI. Patients with head and neck cancer who receive radiotherapy have similar nutritional risks at baseline as those who receive concurrent chemoradiotherapy, with the nutritional risk being particularly high in these patients (28). Similarly, Sun et al. (29) reported that poor nutritional status before radiotherapy and lower albumin levels were linked to treatment-related complications, highlighting the need for early nutritional assessment. Furthermore, our results align with earlier findings showing that pretreatment PNI provides a clinically useful composite measure of nutritional and inflammatory status; however, its components may also reflect underlying systemic conditions. Further, recent data from the NIRT-LC study in older patients with lung cancer undergoing radiotherapy showed that baseline immunonutritional markers such as PNI, GNRI, and CALLY were linked to overall survival, suggesting that pre-treatment immunonutritional evaluation may be prognostically important before radiotherapy (30, 31). Mortality and long-term survival were not assessed in the current cohort, but these findings provide important context for our finding that low baseline PNI correlated with clinically significant weight loss during treatment. These results confirm and complement each other, suggesting that all patients should undergo a simple nutritional evaluation before radiotherapy to detect nutritional problems early.

Our results showed progressive body weight loss during radiotherapy and that the proportion of patients developing clinically significant and critical weight loss, as well as decreased serum albumin and increased PNI, increased over time. Likewise, Zhang et al. (32) found that progressive weight loss during radiotherapy was also a significant factor associated with nutrition-related adverse effects, highlighting the importance of ongoing nutrition monitoring. Our cohort also showed a parallel decrease in albumin levels and an increase in hypoalbuminemia, in line with Prior-Sánchez et al. (33), who found that poor nutritional status during treatment is associated with poor body composition and clinical outcomes. In addition, a significant decrease in PNI and a rise in low PNI are also consistent with the real-world study by Uri et al. (34), which found that a decrease in PNI with progression of nutritional deterioration was correlated with poor prognosis in patients receiving radiotherapy.

Patients with significant weight loss had significantly lower BMI and albumin and PNI scores at baseline, a higher proportion of head and neck and upper gastrointestinal cancers, concurrent chemotherapy, severe toxicity, enteral nutrition, treatment interruption and hospitalization during radiotherapy, suggesting different weight trajectories. Likewise, Jin et al. (35) reported a strong correlation between progressive weight loss and worsening nutrition-impact symptoms and nutritional status over time during radiotherapy. This study also confirmed that treatment-related symptoms to oral intake make a significant contribution to nutritional deterioration, especially for head and neck cancer patients, which is consistent with our findings of increased toxicity and nutritional needs in our marked weight loss group. Similarly, Wang et al. (36) showed that nutritional counseling did not help patients most likely to experience a decline in nutritional intake during radiotherapy, as these patients had worse nutritional intake and clinical outcomes than those with a lower likelihood of declining nutritional intake.

Our cohort showed three distinct PNI trajectories, with greater PNI decline occurring concurrently with greater weight loss, hypoalbuminemia, more severe treatment-related toxicity, more severe oral mucositis, treatment interruption, hospitalization, and radiotherapy non-completion. Likewise, Fujiwara et al. (37) showed that PNI gradually decreases during CRT and can be measured objectively as an indicator of nutritional deterioration. In accordance with our results, Liu et al. (38) found that a decrease in PNI was strongly associated with the development of severe radiation-induced mucositis, especially in patients with head and neck cancer, pointing to a strong link between nutritional deterioration and radiation toxicity. In addition, the clinical findings of poor outcomes in patients with significant PNI reduction corroborate Jiang et al. (39), who identified low PNI as an inflammation-based prognostic marker correlated with poor treatment outcomes and survival. These findings suggest that serial PNI monitoring may help identify concurrent nutritional deterioration during radiotherapy and support timely nutritional assessment and intervention.

In this retrospective cohort, patients who received documented early nutritional support had a lower observed frequency of marked weight loss, hospitalization, and severe treatment-related toxicity, with a tendency toward fewer treatment interruptions. However, because nutritional support was not randomly assigned and its timing may have been influenced by baseline nutritional risk and evolving clinical status, these associations should not be interpreted as evidence of a causal protective effect. Likewise, Leis et al. (11) reported that early nutritional interventions positively impact short-term nutrition and clinical outcomes in HNC patients receiving radiotherapy. Consistent with our results, Zhao et al. (12) noted that clinical guidelines suggest initiating nutritional evaluation and intervention early in the radiotherapy process to reduce nutritional deterioration and support smooth treatment. Similarly, Oppermann et al. (13) found that dietary counseling during radiotherapy is feasible and can improve treatment tolerance and weight maintenance, especially in nutritionally at-risk patients.

While the NIRT-LC study targeted older adults, the findings are relevant to the broader adult oncology population. The tumor’s metabolic demands, radiotherapy toxicity, decreased oral intake, and systemic inflammatory responses are likely to contribute to nutritional deterioration in patients of all ages receiving radiotherapy. Nutrition assessment before treatment and monitoring of nutritional status during radiotherapy may therefore be appropriate for each adult cancer patient population; the intensity and type of nutritional intervention should be tailored to the individual based on tumor site, treatment plan, baseline nutritional status, and changing clinical needs.

Toxicity was common during radiotherapy, with severe toxicity, oral mucositis, dysphagia, anorexia, interruption of treatment, hospitalization, and radiotherapy non-completion being more common in patients with clinically significant weight loss. In a similar study, Choi et al. (40) found that significant weight loss during curative radiotherapy was a significant factor associated with treatment interruption and unplanned hospital admission, raising the clinical risk of nutritional changes. Consistent with our results, early nutritional interventions have been shown to reduce the severity of H&N radiotherapy toxicity and improve tolerance, while worsening nutritional status is associated with an increased risk of severe oral mucositis (41). Overall, these findings indicate that avoiding excessive weight loss during radiotherapy treatment can help reduce treatment-related complications, ensure continuity of treatment, and enhance clinical outcomes.

The secondary severity analysis also showed that severity was graded by the extent of weight loss. Severe toxicity, interruption of treatment, hospitalization and radiotherapy non-completion were the most common among patients with >10% weight loss and the least frequent among patients with <5% weight loss. Thus, while a threshold of ≥5% is clinically relevant for detecting clinically important weight loss, the amount of weight loss may also offer clues about treatment susceptibility and monitoring of nutritional status.

In the primary multivariable model, baseline BMI, planned concurrent chemotherapy, head and neck and upper gastrointestinal cancer site, cancer stage and low baseline PNI were independent factors associated with clinically significant weight loss. The correlation of higher weight loss and more advanced cancer stage could be due to a higher disease burden, systemic inflammatory activity, a higher intensity of care received by patients with more advanced cancer, and/or increased nutritional vulnerability. Importantly, these variables were available before or at radiotherapy initiation and maintained the time-order attribute between baseline and subsequent weight-loss outcomes. The other factors longitudinal PNI trajectory and treatment-associated toxicity were not initially considered as baseline factors because they were evaluated during radiotherapy and may become present at the same time as or because of nutritional deterioration. Functional status represents another potentially important determinant of nutritional vulnerability during radiotherapy. However, ECOG performance status was not consistently documented in the retrospective records and could not be incorporated into the primary multivariable model. Therefore, residual confounding related to baseline functional status cannot be excluded. The results emphasize the need to detect nutritional risk prior to radiotherapy and the need for a nutritional assessment during radiotherapy. Likewise, Leelasawatsuk et al. (42) found that nutritional deficiencies at diagnosis and treatment type were important predictors of critical weight loss during curative radiation therapy. Similarly, it has been shown in previous studies that clinical and treatment related factors and deficiencies at diagnosis are associated with clinically significant weight loss during radiotherapy (43). These clinical and nutritional risk factors may help identify patients for more intensive supportive care and nutritional monitoring. However, this observed link between documented early nutritional intervention and better nutritional outcomes in our cohort should be taken with a grain of salt, as a retrospective study cannot prove causality (44).

The weight loss, frequency of clinically important weight loss, degree of PNI degradation, as well as the severity of treatment-related toxicity were significantly different for patients with head and neck cancer and those with upper gastrointestinal cancer, as shown by our subgroup analysis. These results align with the prospective study by Fange Gjelstad et al. (45), which found that patients undergoing head and neck radiotherapy experienced significant weight loss during treatment due to swallowing problems and reduced food intake. Similarly, Yang et al. (46) found that patients needing intensive nutritional support had greater nutritional decline and immune dysfunction, and concluded that tumor site strongly affected nutritional outcomes and the need for personalized nutrition care. When combined, these results suggest that head and neck and upper gastrointestinal malignancy patients are at the greatest risk of nutritional deterioration during radiotherapy and that they should be considered the top priority for early nutritional assessment and intervention.

### 4.1 Strengths of the study

This study has several important strengths. It contained a comparatively large number of patients (680) with various cancers receiving radiotherapy, thus enhancing the generalizability of the results to other common solid cancers. Baseline to early post-treatment serial assessments of body weight, serum albumin, and the prognostic nutritional index (PNI) allowed evaluation of longitudinal changes in nutritional status, which a single time point could not capture. The study also identified specific weight and PNI trajectory subgroups and explored associations between these subgroups and treatment-related toxicity, hospitalization, treatment interruption, and radiotherapy non-completion. Moreover, the major multivariate logistic regression model was limited to baseline/pre-treatment planning variables, thereby better maintaining the temporal association with clinically significant weight loss. Associations between documented early intervention and nutritional outcomes were observed via clinically relevant data, but this was not of a causal nature as the design was retrospective.

### 4.2 Limitations of the study

However, there are some drawbacks to consider: The retrospective design could have introduced selection bias and may have made causal inferences between nutrition and clinical outcomes more difficult. The study was limited to a single institution and may have limited generalizability. While the study measured nutritional indicators such as body weight, albumin, and PNI, it did not consistently capture other validated measures of nutritional status, including skeletal muscle mass, body composition, dietary intake, inflammatory biomarkers, and patient-reported nutritional symptoms. Differences in tumor biology, treatment options, and nutritional interventions by cancer site may also have influenced the observed outcomes. In addition, the study did not evaluate mortality, long-term survival, quality of life, or functional recovery beyond the first few months after radiotherapy. Therefore, the prognostic implications of the observed nutritional and PNI trajectories beyond treatment tolerance and short-term clinical outcomes remain uncertain. Because patients who discontinued radiotherapy were retained in the analytical cohort, treatment non-completion could be evaluated as a clinically relevant outcome; however, incomplete post-discontinuation nutritional measurements may have introduced differential follow-up and should be considered when interpreting longitudinal changes at later assessment points.

One drawback was the incomplete recording of ECOG performance status in the retrospective clinical records. A consistent functional-status measure was not available; therefore, ECOG was not included in the main multivariable analyses. This means residual confounding due to baseline functional impairment is possible. Differences in staging systems between tumor types and disease characteristics may have left residual heterogeneity in the primary multivariable model, despite including cancer stage.

Furthermore, some potential clinical confounders possibly affecting serum albumin, lymphocyte and PNI levels were not systematically recorded or consistently available in the retrospective dataset, such as hepatic dysfunction and other systemic diseases. The differences in the nutritional indices or inflammatory parameters may be attributed to these and could not be explained in this analysis. Thus, the observed relationships between baseline albumin and PNI must be considered and interpreted in the context of the possibility of residual confounding. There was no systematic record keeping of when nutritional consultation and intervention was offered or received in the retrospective records. Thus a comparison of early versus delayed nutrition intervention and subsequent weight outcomes was not possible. Furthermore, nutritional support was clinically indicated and could have been preferentially given to those patients who had more nutritional risk or treatment-related symptoms, which may result in confounding by indication. As such, the relationship between reported nutrition support and weight outcomes should be considered as a treatment effect of nutritional support rather than a causal relationship.

Furthermore, radiotherapy regimens varied across tumor sites and indications, which meant calendar time between longitudinal nutritional assessments varied. Analyses were performed consistent with the pre-specified treatment phases but this could have affected the size and timing of the nutritional shifts observed. A smaller subset of the patients with >10% weight loss was analyzed for secondary weight loss severity. This group was adequately sampled to make description comparisons, but the smaller sample sizes restrain the precision of the estimates of individual treatment-related outcomes. Thus, the analysis of findings of the three categories of severity should be taken as exploratory and should be interpreted in conjunction with the main analysis of ≥5% weight loss.

### 4.3 Future recommendations

Multicenter studies in larger, more varied patient cohorts and treatment settings should replicate these results. Research should include comprehensive nutritional assessment tools such as body composition analysis, dietary intake, inflammatory markers, and patient-reported outcomes to better define nutritional deterioration in the context of radiotherapy. Randomized controlled trials of individualized nutrition interventions started before treatment are warranted to assess whether these interventions promote weight maintenance, reduce treatment-related toxicity and hospitalization, and improve treatment completion and survival. Furthermore, predictive models based on longitudinal nutritional trajectories and artificial intelligence could help identify patients at high nutritional risk early, allowing optimisation of nutritional care during radiotherapy.

## 5 Conclusion

Weight loss and nutritional deterioration were prevalent in the body during radiotherapy. Baseline nutritional vulnerability and treatment planning characteristics were associated independently with clinically significant weight loss, while deterioration in PNI was associated with concurrent nutritional deterioration and more treatment-related toxicity, hospitalization, treatment interruption, and radiotherapy non-completion. Documented nutritional support during the early treatment period was associated with more favorable weight and treatment outcomes; however, the timing of intervention was not consistently documented and these observational associations should not be interpreted as causal. The results indicate the need for an ongoing (pre and during) nutritional assessment, and a periodic (every session) nutritional monitoring of the patient during radiotherapy, to guide the selection of suitable patients for timely multidisciplinary supportive care.

## Data Availability

The raw data supporting the conclusions of this article will be made available by the authors, without undue reservation.
